# Biological and transcriptional studies reveal VmeL is involved in motility, biofilm formation and virulence in *Vibrio parahaemolyticus*

**DOI:** 10.3389/fmicb.2022.976334

**Published:** 2022-08-09

**Authors:** Peng-xuan Liu, Xiao-yun Zhang, Quan Wang, Yang-yang Li, Wei-dong Sun, Yu Qi, Kai Zhou, Xian-gan Han, Zhao-guo Chen, Wei-huan Fang, Wei Jiang

**Affiliations:** ^1^Shanghai Veterinary Research Institute, Chinese Academy of Agricultural Sciences (CAAS), Shanghai, China; ^2^College of Veterinary Medicine, Nanjing Agricultural University, Nanjing, China; ^3^Shenzhen Institute of Respiratory Diseases, The First Affiliated Hospital (Shenzhen People’s Hospital), Shenzhen, China; ^4^Institute of Preventive Veterinary Medicine and Zhejiang Provincial Key Laboratory of Preventive Veterinary Medicine, Zhejiang University, Hangzhou, China

**Keywords:** *Vibrio parahaemolyticus*, VmeL, swarming, biofilm, RNA-sequencing, pathogenicity

## Abstract

*Vibrio parahaemolyticus* is a marine pathogen thought to be the leading cause of seafood-borne gastroenteritis globally, urgently requiring efficient management methods. *V. parahaemolyticus* encodes 12 resistance/nodulation/division (RND) efflux systems. However, research on these systems is still in its infancy. In this study, we discovered that the inactivation of VmeL, a membrane fusion protein within the RND efflux systems, led to reduction of the ability of biofilm formation. Further results displayed that the decreased capacity of Congo red binding and the colony of Δ*vmeL* is more translucent compared with wild type strains, suggested reduced biofilm formation due to decreased production of biofilm exopolysaccharide upon *vmeL* deletion. In addition, the deletion of *vmeL* abolished surface swarming and swimming motility of *V. parahaemolyticus*. Additionally, deletion of *vmeL* weakened the cytotoxicity of *V. parahaemolyticus* towards HeLa cells, and impaired its virulence in a murine intraperitoneal infection assay. Finally, through RNA-sequencing, we ascertained that there were 716 upregulated genes and 247 downregulated genes in Δ*vmeL* strain. KEGG enrichment analysis revealed that quorum sensing, bacterial secretion systems, ATP-binding cassette transporters, and various amino acid metabolism pathways were altered due to the inactivation of *vmeL*. qRT-PCR further confirmed that genes accountable to the type III secretion system (T3SS1) and lateral flagella were negatively affected by *vmeL* deletion. Taken together, our results suggest that VmeL plays an important role in pathogenicity, making it a good target for managing infection with *V. parahaemolyticus.*

## Introduction

*Vibrio parahaemolyticus* is a gram-negative halophilic bacterium, primarily found in warm marine or estuarine environments (Broberg et al., 2011). *V. parahaemolyticus*, discovered almost 70 years ago, has been identified as a leading cause of foodborne illness worldwide, and could lead septicemia through wound infection (Su and Liu, 2007; Ghenem et al., 2017). In recent years, the rate of *V. parahaemolyticus* infection has increased in many countries, including the United States and China (Banu et al., 2018; Lei et al., 2020). *V. parahaemolyticus* exerts its virulence through diverse factors (Li et al., 2019). Of these, T3SSs are prominent virulence factors, and can inject diverse effectors into eukaryotic cells through a transmembrane apparatus. However, some *V. parahaemolyticus* isolates can induce acute gastroenteritis without T3SS2 and hemolysin genes (Ottaviani et al., 2012). This indicates alternative mechanisms that contribute to the pathogenicity of *V. parahaemolyticus*.

As a ubiquitous marine bacterium and human pathogen, *V. parahaemolyticus* has evolved several motility mechanisms to promote colonization of deep sea vents or the bodies of animals. *V. parahaemolyticus* exhibits multiple cell types that are appropriate for life under different circumstance (McCarter, 1999). Swimmer cells synthesize a single polar flagellum for adaption to life in liquid environments, and swarmer cells propelled by many proton-powered lateral flagella, for movement through highly viscous environments, colonize surfaces, and form multicellular communities which sometimes display highly periodic architecture (Boles and McCarter, 2002; Wadhwa and Berg, 2021). Motility is essential for adaptability, survival, and infection of the human host (Taw et al., 2015). In addition, *V. parahaemolyticus* could improve its survival in adverse surroundings through biofilm formation, enhancing antibiotic resistance and leading to the development of intractable infections (Yildiz and Visick, 2009; Harshey and Partridge, 2015; Hall and Mah, 2017; Hotinger et al., 2021).

Various efflux pumps are important contributors to antibiotic resistance in microbes (Uddin et al., 2021). Resistance/nodulation/division (RND) efflux systems are ubiquitous in Gram-negative bacteria, and are linked to antimicrobial resistance (Alvarez-Ortega et al., 2013). RND efflux systems consist of an inner membrane protein, two membrane fusion proteins and an outer membrane pore protein (Alav et al., 2021). The three core constituents operate collectively to efflux several substrates from the cytoplasm and periplasm to the external environment. Interestingly, an increasing number of studies have demonstrated that RND efflux systems have many functions in diverse phenotypes including metabolism, biofilm, and virulence. For instance, the loss of six efflux systems in *V. cholerae* represses the production of several virulence factors (Bina et al., 2018). The inactivation of *abeD*, which encodes a membrane transporter, reduced virulence toward a nematode model in *Acinetobacter baumannii* (Srinivasan et al., 2015). In addition, the inner membrane protein AdeJ could negatively influenced surface motility and biofilm formation in *A. nosocomialis* (Knight et al., 2018). Thus, previous studies have suggested that RND efflux systems in bacteria were involved not only in antimicrobial resistance, but also in biological and pathophysiological processes.

Twelve RND-type efflux transporter genes in *V. parahaemolyticus* have been estimated in a previous study, and their finding indicated that resistance nodulation cell division-type efflux transporters contribute not only to intrinsic resistance but also to exerting the virulence of *V. parahaemolyticus* (Matsuo et al., 2013). However, little is known about the contribution of RND efflux systems to *V. parahaemolyticus* pathogenicity and host adaptation. The membrane fusion proteins, also known as the periplasmic adaptor proteins, play central role in the primacy of the determinants of substrate specificity and the assemble of the RND efflux systems (Alav et al., 2021). Despite their central importance for the efflux process, the function of membrane fusion proteins remains least-well understood. Therefore, in this study, the biological contributions of membrane fusion protein VmeL in surface motility, biofilm formation, and virulence of *V. parahaemolyticus* were further examined. Our findings offer insight into the roles of this protein, and can serve as a basis for the control of *V. parahaemolyticus* contamination and infection.

## Materials and methods

### Bacterial strains, plasmids, and growth conditions

The wild-type (WT) strain *V. parahaemolyticus* SH112 (GenBank: JACYGZ000000000.1) stocked in the China General Microbiological Culture Collection Center under the accession number CGMCC 1.90013. *V. parahaemolyticus* was cultured in MLB (Lysogeny broth with 2% sodium chloride) at 37 °C with constant shaking at 180 rpm. *Escherichia coli* CC118 λ*pir* was cultivated in LB medium at 37 °C with shaking at 180 rpm; 70 μg/mL kanamycin and 10 μg/mL chloramphenicol were supplemented for specific plasmids. Table 1 displays all WT and derivative strains of *V. parahaemolyticus* and *E. coli*.

**TABLE 1:** Bacterial strains and plasmids.

Strains or plasmids	Description	Resource
*V. parahaemolyticus* strains		
Wild type, WT	*V. parahaemolyticus* SH112	Lab collection
Δ*vmeL*	Mutation in *vmeL* gene of strain SH112	This study
CΔ*vmeL*	Δ*vmeL* strain with a plasmid pMMB-*vmeL*	This study
*E. coli strains*		
HB101	Strains with a helper plasmid pRK2013	Lab collection
CC118 λ*pir*	Λpir lysogen of CC118 Δ (*ara*-*leu*) *ara*D Δ*lacX74 galE galK phoA20 thi-1 rpsE rpoB argE* (Am) *recA1*	Yu et al., 2012
Plasmids		
pMMB207	RSF1010 derivative, *Inc*Q *lacI* q Cm^r^ P*tac ori*T	Morales et al., 1991
pRK2013	Helper plasmid, for gene insertion in chromosome; kana^r^	Figurski and Helinski, 1979
pYAK1	A suicide vector with ori R6K *sacB*; Cm^r^	Ono et al., 2006
pYAK1-*vmeL*	the upstream and downstream regions flanking *vmeL* is digested into suicide vector pYAK1 for construct gene-deleted strain	This study
pMMB207-*vmeL*	VmeL expression plasmid on pMMB207 driven by tac promoter	This study

HeLa cells were cultured in DMEM (Gibco, Thermo Fisher Scientific, Waltham, MA, United States) with 10% fatal bovine serum (FBS, Gibco) and 100 units/mL of streptomycin and penicillin (Invitrogen, Thermo Fisher Scientific) at 37°C with 5% CO_2_ humidity.

### Construction of the *vmeL* mutant and complemented strains

All primers used for the strain and plasmid construction in this study are shown in Table 2. The deletion mutant was constructed through homologous recombination, as previously described (Lian et al., 2021). First, *vmeL*-A/B and *vmeL*-C/D were utilized to amplify the upstream and downstream regions flanking *vmeL*, respectively. A fragment comprised of flanking sequences was generated using these two fragments as templates, and connected with the suicide plasmid pYAK1, yielding the recombinant plasmid pYAK1-*vmeL*. This pYAK1-*vmeL* was transferred into *V. parahaemolyticus* SH112 through triparental conjugation. After double crossover recombination, the successful mutant was designated as Δ*vmeL.*

**TABLE 2:** Primers were used to construct Δ*vmeL* and CΔ*vmeL.*

Primers	Sequence (5′–3′)	Restriction Site^a^	Target Gene
*vmeL*-A	CGC**GGATCC**TTGAGGTAAATGTTTGGCACAG	*Bam* HI	Updream region of *vmeL* ORF (444 bp)
*vmeL*-B	CAAAAATCCTTTTTTATTGTTG		
*vmeL*-C	AAAAAAGGATTTTTGGTAGGAGTTTGTCATGGAAG		Down dream region of *vmeL*(611 bp)
*vmeL*-D	TCC**CCCGGG**ACTTACGCAGCACGAGGG	*Sma* I	
*vmeL*-E	CGGCTAAAGAACGAGTGT		Wide type: 2,648 bp
*vmeL*-F	TTCTTCGGTAAAGCCATC		Mutant: 1,490 bp
pMMB207-*vmeL*-F	CGC**GGATCC**ATGTTAGCAAAACCCATAGTA	*Bam* HI	A fragment for complementation of *vmeL* (1,177 bp)
pMMB207-*vmeL*-R	ACAT**GCATGC**TTACTGTACGACGGTTTCGGA	*Sph* I	
*sacB*-F	ACGGCACTGTCGCAAACTATA		
*sacB*-R	TTCCGTCACCGTCAAAGAT		

^a^Restriction sites are bolded.

The complemented strain was constructed similarly to the construction of the mutant using pMMB207. Briefly, the total *vmeL* open reading frame was amplified utilizing the primer pMMB207-*vmeL*-F/R. The purified PCR product was linked to the pMMB207 plasmid with *BamHI* and *SphI* enzymes, resulting in the plasmid pMMB207-*vmeL*. Then, the plasmid was transformed into Δ*vmeL* using a method similar to that used for the Δ*vmeL* mutant. The complemented strain was designated as CΔ*vmeL.*

### Analysis of growth curves

The overnight cultures were diluted at 1:100 in fresh MLB medium or in DMEM medium containing 10% FBS and incubated at 37°C. Aliquots (200 μL) were transferred to clear 96-well plates every hour, and the optical density of the culture at 600 nm (OD_600_) was assessed utilizing a spectrophotometer (Thermo Fisher Scientific) at 1 h intervals for 10∼14 h. In the following experiments, the cells were utilized at logarithmic phage (OD_600_ = 0.2 ± 0.02) or about 1 × 10^8^ cells per mL.

### Antimicrobial susceptibility test

The minimum inhibitory concentrations (MICs) of several antibiotics were examined in Mueller-Hinton (MH) broth, as described previously (Matsuo et al., 2007). Briefly, MICs were processed in MH broth containing antibiotics in a two-fold dilution series. Bacteria were cultured in the test medium at 37°C for 24 h. Each compound’s MIC is defined as the lowest concentration that prevents visible growth.

### Crystal violet staining assays

Crystal violet staining assays were tested as previously described (Zhang et al., 2021a) with some modifications. Overnight cultures were diluted at 1:100 in fresh MLB medium, 200 μL aliquots of the test organism suspension were cultured in 96-well microtiter dishes (Costar, 42592), and then incubated for 48 h at 30 °C without shaking. Then, each well was rinsed with phosphate-buffered saline (PBS), and 250 μL of 0.1% crystal violet solution was added to each well for 15 min at 20–25°C. The wells were rinsed with PBS. 200 μL of 95% (v/v) ethanol was used to dissolve crystal violet, and the opacity of each wells was assessed by measuring OD_595_.

### Colony morphology and congo red binding assays

Both assays were performed as described formerly (Enos-Berlage et al., 2005; Zhang et al., 2021a). For morphology analysis, overnight cultures were diluted at 1:100 in fresh MLB medium, then, the logarithmic bacterial culture was homogenized, and 2 μL aliquots were spotted on heart infusion medium (HI) (ELITE Biotech, Shanghai, China) plates containing 2% NaCl and 2% agar, then incubated at 37°C for at least 48 h. For the CR binding assay, the mixed bacterial culture was spotted on CR plates [HI plates added 80 ug/ml Congo red (Yuanye Bio-Technology, Shanghai, China)], and then incubated at 30°C for 8 d. Images of each strain were recorded every two days.

### Motility assays

Motility assays were conducted as described previously (Whitaker et al., 2014), with some modifications. Briefly, overnight cultures were diluted at 1:100 in fresh MLB medium and swimming motility assays were performed using 2 μL of logarithmic bacterial culture spotted onto surface of LB plates containing 1% NaCl and 0.3% agar. Images of each plate were captured after incubating for 4–5 h at 37°C. Swarming motility assays were performed similarly but on HI plates containing 2% NaCl and 1.5% agar. Strains were incubated at 30°C for 15–20 h before images were taken. For both motility assays, the colony diameters were measured and recorded.

### Transmission electron microscopy of flagella

Transmission electron microscopy of the lateral flagella was performed as previously described (Gu et al., 2019), with some modifications. All strains were cultured on HI agar plates for 15 h, rinsed lightly with 0.01 M PBS. Subsequently, 5 μL of bacterial suspension was dropped onto the copper grid and left until the mesh was dry enough to cover. Then, samples were observed by TEM (Tecnai G2 Spirit, FEI Company, Hillsboro, OR, United States).

### Infection of HeLa cells

According to a previous report (Tandhavanant et al., 2018), assays of infection of HeLa cells were conducted. HeLa cells were plated in 24-well dishes at a density of 1.5 × 10^5^ per cell, grown for 12 h, and then infected with WT, Δ*vmeL*, and CΔ*vmeL V. parahaemolyticus* at MOI of 10 for 2 h. After 2 h f co-incubation, the cells were rinsed with PBS and fixed with 4% paraformaldehyde in PBS for 15 min at room temperature. 0.5% Triton-X was used to permeabilize the cells for 10 minat room temperature. Then, the cells were probed with rhodamine-phalloidin (SBS Genetech, Shanghai, China) to stain F-actin and DAPI (Beyotime, Shanghai, China) to highlight HeLa cell DNA. Images were captured using an inverted fluorescence microscope (Axio observer Z1, ZEISS).

### Lactate dehydrogenase release assay

HeLa cells were plated in 96-well dishes at 2 × 10^4^ cells per well, cultured for 12 h, and over-night bacterial suspensions were diluted in fresh MLB medium and cultivated to the logarithmic phase. The next day cells were infected with WT, Δ*vmeL*, and CΔ*vmeL V. parahaemolyticus* in four replicates at a multiplicity of infection (MOI) of 10. LDH release was measured using a CytoTox96 kit (Promega, Madison, WI, United States), and absorbance was measured using a spectrophotometer (Multiskan Go, Thermo Fisher Scientific).

### Animal infection experiments

Animal infection experiments were conducted as previously described (Li et al., 2022). Over-night bacterial suspensions were diluted in fresh MLB medium and cultivated to the logarithmic phase. The logarithmic bacterial culture of each strain was washed three times with PBS. Then, 1 10^7^ CFU of each strain was inoculated intraperitoneally into female Institute of Cancer Research mice at three to four weeks of age. After infection, the symptoms of inoculated mice and number of deaths were recorded. The Animal Ethics Committee of the Shanghai Veterinary Research Institute, Chinese Academy of Agricultural Sciences approved all animal infection studies (no. SYXK<HU > 2020-0027).

### RNA-seq and data processing

When cultured strains reached the exponential phase in MLB medium, total RNA was isolated using Bacteria RNA Extraction Kit (Vazyme, Nanjing, China). Three independent biological samples were conducted, then cDNA libraries were prepared utilizing a customized procedure (Sangon Biotech, Shanghai, China). Low-quality counts were removed utilizing the R package DESeq2 and to filter differential expression in accordance with the criteria of an absolute log2 fold-change value of >1.5, and a *p*-value of < 0.01. To analyze the differential expression of genes between the WT and Δ*vmeL* strains, differentially expressed genes (DEGs) were processed through Kyoto Encyclopedia of Genes and Genomes (KEGG) database using the ClusterProfiler package, and the *Q*-value was regarded as a screening criterion.

### Quantitative reverse-transcription PCR analysis

Quantitative reverse-transcription PCR was used to examined the transcriptional levels of different genes in the WT and mutant strains. Overnight cultures of WT, Δ*vmeL*, and CΔ*vmeL V. parahaemolyticus* were adjusted to an OD_600_ of 0.2, and 2.5 μL aliquots were spread separately onto swarming plates. Cells were collected from swarming plates after 15 h of cultivation, and were suspended in 1 mL of TRIzol (Yu et al., 2019). Planktonic bacteria were cultivated in liquid MLB medium to the logarithmic phase with shaking at 37°C, and the swarming cells were collected. Then, total RNA was isolated from each sample following manufacturer protocol (Bacteria RNA Extraction Kit, Vazyme, Nanjing, China). RNA was reverse transcribed to cDNA. cDNA was utilized as a template for quantitative qRT-PCR. For each gene, reactions were performed for three RNA samples and each reaction was performed in triplicate. The transcriptional level was quantified using the 2^–ΔΔ*Ct*^ method using the housekeeping gene *gapA* (Genbank: BAC61233.1) for normalization (Livak and Schmittgen, 2001). The primers for qRT-PCR are provided in Supplementary Table 1.

### Statistical analysis

Graphpad Prism was used for data analysis. Images were processed using Microsoft Office PowerPoint. Basic Local Alignment Search Tool (BLAST) analysis was conducted using the available tool^1^. The qRT-PCR results were analyzed using a two-way analysis of variance (ANOVA) and a one-way ANOVA was performed for biofilm formation, motility, and cytotoxicity. The survival percentage was analyzed with Gehan-Breslow-Wilcoxon and log rank tests.

## Results

### VmeL is highly conserved in *Vibrio parahaemolyticus* and several *Vibrio* species

*Vibrio parahaemolyticus* strain SH112 was used to amplify the nucleotide sequence of membrane fusion protein VmeL, and the sequence of *vmeL* (GenBank: OL347638) displayed 99.83% identity with that of *V. parahaemolyticus* RIMD2210633 (GenBank: BA000032.2). BLASTn analyses indicated that the nucleotide sequence similarity between a total of 143 *V. parahaemolyticus* isolates ranged from 97.84 to 99.91% (Supplementary Figure 1A). Furthermore, a BLASTp search against GenBank suggested that VmeL in *V. parahaemolyticus* SH112 shared 94.03, 92.47, 92.99, 93.51, and 92.21% similarity with orthologous proteins in *V. diabolicus* (GenBank: WP_005392685.1), *V. harveyi* (GenBank: WP_005441462.1), *V. alginolyticus* (GenBank: EGQ8497134.1), *V. chemaguriensis* (GenBank: WP_225460886.1), and *V. rotiferianus* (GenBank: WP_038882841.1), respectively (Supplementary Figure 1B). These findings show that VmeL is an evolutionarily conserved membrane fusion protein found in *V. parahaemolyticus* and several *Vibrio* species.

### VmeL does not affect the growth capacity of *Vibrio parahaemolyticus*

The resulting null mutant strain Δ*vmeL* and the complemented strain CΔ*vmeL* were examined by PCR and qRT-PCR (Supplementary Figure 2). No significant distinction in growth rate was identified between WT, Δ*vmeL*, and CΔ*vmeL* strains in MLB medium or in DMEM medium containing 10% FBS (Supplementary Figure 3).

### VmeL of *Vibrio parahaemolyticus* does not influence antimicrobial resistance

To test whether the deletion of *vmeL* could influence antimicrobial resistance in *V. parahaemolyticus*, we performed an antimicrobial susceptibility test. The results showed that loss of *vmeL* in *V. parahaemolyticus* had no significant influence on response to any of the tested antimicrobial compounds. The MICs were identified for streptomycin (22.25 μg/mL), cefalotin sodium (3.75 μg/mL), gentamicin (6.25 μg/mL), ampicillin (> 100 μg/mL), florfenicol (0.94 μg/mL), chloramphenicol (0.46 μg/mL), tetracycline (0.36 μg/mL), kanamycin (17.5 μg/mL).

### VmeL contributes to biofilm formation

**FIGURE 1:**
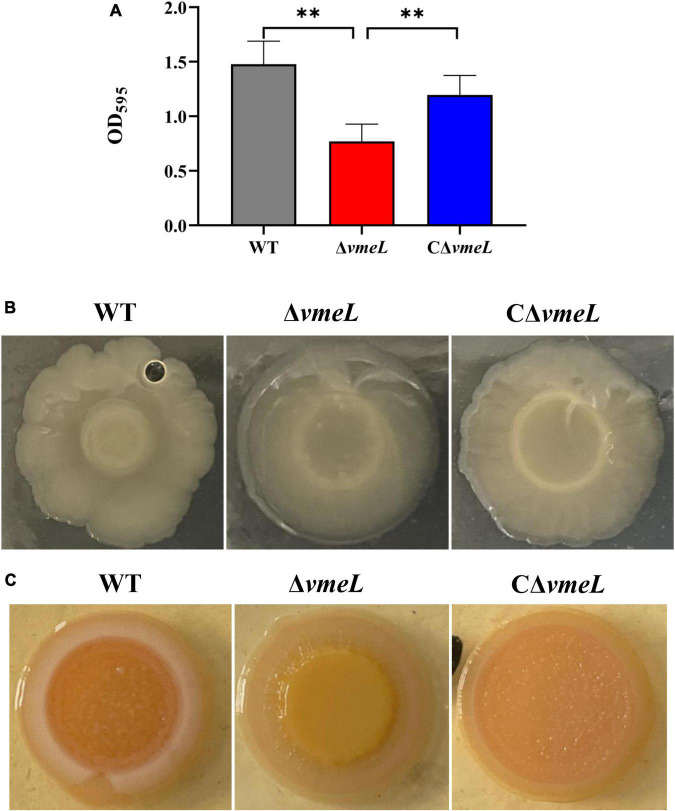
VmeL is required for biofilm formation by *Vibrio parahaemolyticus*. **(A)** Optical density at 595 nm (OD_595_) for crystal violet biofilm assays. Data shown are the mean of six replicates. **(B)** Colony morphology of strains cultivated on heart infusion plates for at least 48 hours. **(C)** Strains cultivated on Congo Red plates for eight days and photographed. WT, wild-type; Δ*vmeL*, *vmeL* deletion mutant; CΔ*vmeL*, deletion mutant complemented strain. ** indicates statistical significance at *P* < 0.01.

To assay whether VmeL could affect the ability of biofilm formation in *V. parahaemolyticus*, the crystal violet staining assay was performed. The results showed that biofilm formation by Δ*vmeL* was significantly reduced compared to that of the WT strain (*P* < 0.01), while the capacity of biofilm formation was restored in complemented stains (Figure 1A). Since colony morphology is closely related to the synthesis of abundant exopolysaccharides (Enos-Berlage et al., 2005), we next examined whether the loss of *vmeL* could influence the colony morphology of *V. parahaemolyticus*. Δ*vmeL* produced more translucent colonies than those of the WT strain, while CΔ*vmeL* produced opaque colonies similar to those of the WT strain (Figure 1B). In addition to colony morphology, the capacity of CR binding is closely related with the content of biofilm exopolysaccharide in *V. parahaemolyticus* (Enos-Berlage et al., 2005). At the center of the colony, the Δ*vmeL* strain displayed a smoother and lighter red color than that of the WT strain under our experimental conditions (Figure 1C). These observations indicated that VmeL positively modulates biofilm formation.

### Deletion of *vmeL* abolishes the swarming and swimming motility of *Vibrio parahaemolyticus*

**FIGURE 2:**
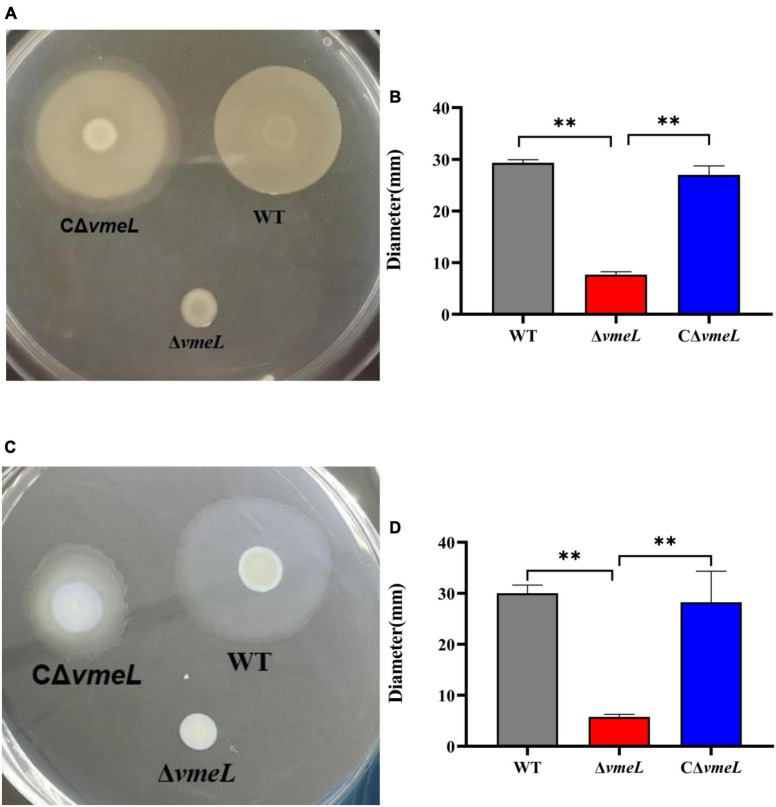
VmeL is required for surface motility of *Vibrio parahaemolyticus*. **(A)** Swarming motility was tested on swarming plates (heart infusion medium with 2% NaCl and 1.5% agar) at 30°C, and images were taken after 18 h. **(C)** Swimming motility was tested on swimming plates (Lysogeny broth supplemented with 1% NaCl and 0.3% agar) at 37°C, and images were taken after 4–5 h. **(B,D)** Representation of the swarming and swimming separately colony diameters, measured in three independent assays. WT, wild-type; Δ*vmeL*, *vmeL* deletion mutant; CΔ*vmeL*, deletion mutant complemented strain. ^**^ indicates statistical significance at *P* < 0.01.

To determine whether VmeL affects motility, surface swarming and swimming motilities of the Δ*vmeL* mutant were compared to those of the WT strain. The Δ*vmeL* strain did not have the capacity of surface swarming and swimming motility compared to the WT strain (Figures 2A,C). the colony diameter of WT and Δ*vmeL* on swarming plates were 29.3 ± 0.7 and 7.6 ± 0.6 mm respectively, and the colony of CΔ*vmeL* was 27 ± 2 mm (Figure 2B). In addition, the colony diameters of WT and Δ*vmeL* on swimming plates were 30 ± 2 and 5.75 ± 0.75 mm, respectively, and the colony of CΔ*vmeL* was 28.25 ± 5.25 mm (Figure 2D). The complemented strains restored the surface motility. Moreover, there is an extra zone of swarming for the complemented strain and the swimming colonies of complemented strains displayed irregular margins. These results indicated that the inactivation of *vmeL* had negative effects both on swarming motility and swimming motility in *V. parahaemolyticus*.

### Inactivation of *vmeL* inhibits the formation of lateral rather than polar flagella

**FIGURE 3:**
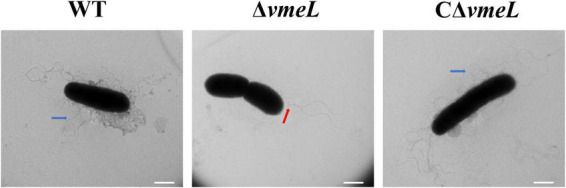
Transmission electron microscope (TEM) images of *Vibrio parahaemolyticus* flagella. Representative images of flagella in WT, Δ*vmeL* and CΔ*vmeL* were observed by TEM and shown. Scale bar = 1 μm. WT, wild-type; Δ*vmeL*, *vmeL* deletion mutant; CΔ*vmeL*, deletion mutant complemented strain.

To examine lateral flagella morphology of WT, Δ*vmeL*, and CΔ*vmeL* strains, we collected samples from swarming agar plates. TEM results showed that the lateral flagella of Δ*vmeL* cells were absent and the polar flagella (red arrow) were still present, whereas each WT and CΔ*vmeL* cell exhibited multiple peripheral flagella (blue arrows) and a polar flagellum (Figure 3). The presence of polar flagella among these cells suggested that swimming inhibition of Δ*vmeL* was due to flagellar malfunction rather than a default in flagellum biosynthesis.

### Deletion of *vmeL* lessens the cytotoxicity of *Vibrio parahaemolyticus* towards HeLa cells

**FIGURE 4:**
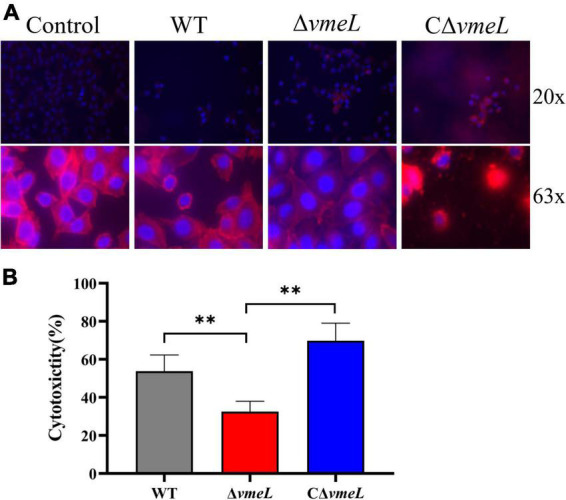
VmeL-mediated virulence toward HeLa cells. **(A)** Representative images showed HeLa cells infected with WT, Δ*vmeL*, and CΔ*vmeL* strains, and one uninfected control group. DNA is shown in blue, and actin is in red. The top panels were photographed at 20 x magnification and the bottom panels were photographed at 63 x magnification. **(B)** Lactate dehydrogenase released by HeLa cells infected with WT, Δ*vmeL*, and CΔ*vmeL* strains of *Vibrio parahaemolyticus* were tested at 2 h. Data represent the means of six replicates. WT, wild-type; Δ*vmeL*, *vmeL* deletion mutant; CΔ*vmeL*, deletion mutant complemented strain. ^**^ indicates statistical significance at *P* < 0.01.

To further investigate the role of VmeL, we next assessed whether the WT and Δ*vmeL* strains exhibited different levels of cytotoxicity towards HeLa cells. Morphological changes induced in HeLa cells by each strain were tested through staining with DAPI and phalloidin. The results showed that the number of cells on coverslips changed considerably after co-incubation with Δ*vmeL*, WT and CΔ*vmeL*. Compared with WT and CΔ*vmeL*, more HeLa cells remained on coverslips after incubating with Δ*vmeL*. As showed in Figure 4A, compared with untreated cells, the infected cells displayed morphological changes including rounding and lysis. Compared with WT, the cells incubated with Δ*vmeL* displayed lesser morphological changes, and the cells incubated with CΔ*vmeL* displayed a significant cytoplasmic lysis phenomenon. These phenomenon suggested Δ*vmeL* strains exhibited lower cytotoxicity towards HeLa cells than did other strains. Further LDH assays sustained our conjecture. After co-incubation for 2 h, a significant decrease in LDH release was observed in the HeLa cells co-incubated with Δ*vmeL* strains compared with those incubated with the WT strain (Figure 4B). Together, these results suggested that VmeL affected the virulence of *V. parahaemolyticus* at the cellular level.

### Inactivation of *vmeL* lessens the virulence of *Vibrio parahaemolyticus* towards mouse

**FIGURE 5:**
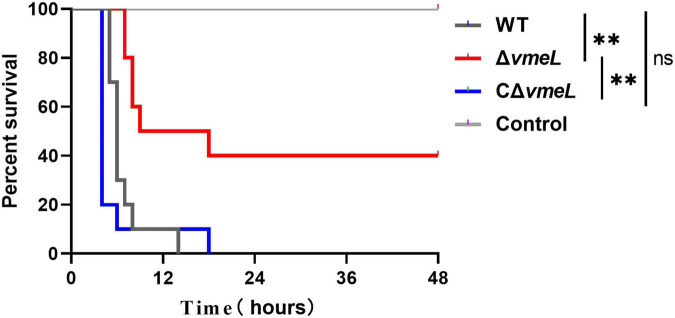
Institute of Cancer Research female mice (n = 10) were infected through intraperitoneal injection with WT, Δ*vmeL*, or CΔ*vmeL* strains of *Vibrio parahaemolyticus*. Mouse mortality was recorded at the times indicated. WT, wild-type; Δ*vmeL*, *vmeL* deletion mutant; CΔ*vmeL*, deletion mutant complemented strain. ** indicates statistical significance at *P* < 0.01.

We next aimed to determine whether VmeL of *V. parahaemolyticus* is involved in virulence. As seen in Figure 5, mice infected with the WT strain exhibited zero survival, while those infected with the Δ*vmeL* showed 40% survival 48 h after infection. In addition, the mice infected with CΔ*vmeL* exhibited zero survival. Meanwhile, all negative control mice survived. After 24 h of infection, no further lethal symptoms were seen in the mice infected with the *vmeL* deletion mutant. These results suggested that VmeL in *V. parahaemolyticus* was involved in pathogenesis towards mouse.

### VmeL influences various expression profiles of *Vibrio parahaemolyticus*

**FIGURE 6:**
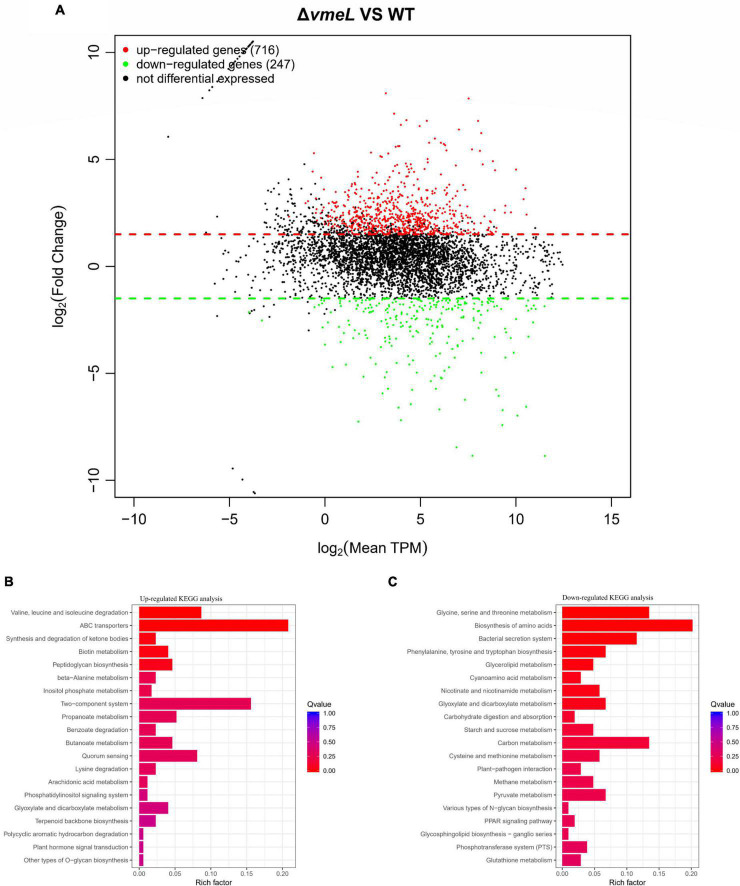
Transcriptomic analysis between Δ*vmeL* and WT strains of *Vibrio parahaemolyticus*. **(A)** MA plot shows the DEGs of Δ*vmeL* and WT. DEGs with log2FC > 1.5 are denoted in red, and DEGs with log2FC < 1.5 are denoted in green (*P* < 0.01). TPM indicates transcripts per kilobase of exon model per million mapped reads. **(B,C)** KEGG pathway enrichment analysis for Δ*vmeL* and WT strains. The color of the bar corresponds to different ranges of Q values (adjusted *P* values). WT, wild-type; Δ*vmeL*, *vmeL* deletion mutant; CΔ*vmeL*, deletion mutant complemented strain.

To identify genes affected by VmeL, we performed transcriptome analysis of Δ*vmeL* cells using RNA-seq. Total RNA was isolated from Δ*vmeL* and WT strains cultured in MLB medium. In three biological replicates, the transcriptomes of the WT strain were compared with those of the Δ*vmeL* strain (*P* < 0.01, log2| FoldChange| > 1.5). RNAseq analysis showed the transcripts of the other 11 RND-efflux systems, as well as the outer membrane protein *vpoc*, were not influenced by the loss of *vmeL*. However, RNA-seq identified 963 genes that were differentially expressed in Δ*vmeL* (Supplementary Material). Of these DEGs, 716 genes were upregulated in Δ*vmeL* when compared to WT (Figure 6A and Supplementary Figure 4), indicating that many functions were repressed through mechanisms involving VmeL.

To further identify the functions of these DEGs, transcriptional profiles were analyzed using the KEGG database (Figures 6B,C). Twelve genes classified as part of the bacterial secretion system (ko03070) pathway were downregulated. Interestingly, all of these genes were related to T3SS1. In addition, a total of 36 ATP-binding cassette (ABC) transporter genes (ko02010) were upregulated in the *vmeL* mutant. 27 genes involved in two-component systems (ko02020) were upregulated, and 14 quorum sensing genes were upregulated. Furthermore, a large number of DEGs were enriched in the microbial metabolism pathways. For instance, 15 genes involved in valine, leucine, and isoleucine degradation (ko00280) were upregulated. Taken together, the results of transcriptional profiling suggested that VmeL is associated with many metabolic and virulence processes in *V. parahaemolyticus*.

### Transcriptional levels of the T3SS1 and flagellar gene in Δ*vmeL*

**FIGURE 7:**
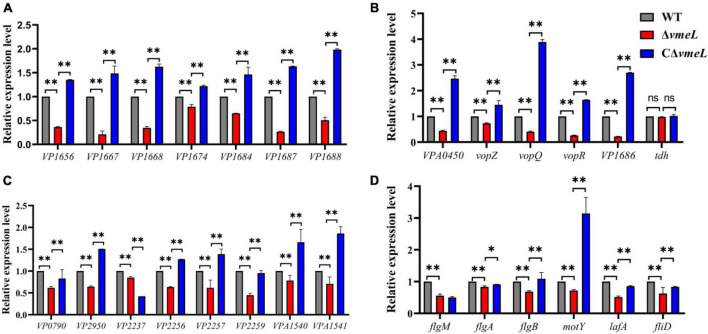
Quantitative reverse transcription-PCR verification of target genes in strains WT, Δ*vmeL*, CΔ*vmeL*. The transcriptional levels of **(A)** T3SS1 structural genes, **(B)** T3SS1 effector genes and **(C)** flagellar-related genes were tested using the templates that collected from strains incubated in liquid medium. **(D)** The first gene of every operon was chosen to verify the transcriptional levels of lateral flagella genes in Δ*vmeL* as compared to those in the wild type (WT), and the templates that collected from strains incubated on swarming plates. WT, wild-type; Δ*vmeL*, *vmeL* deletion mutant; CΔ*vmeL*, deletion mutant complemented strain. ** indicates statistical significance at *P* < 0.01. ns, no significant difference.

Transcriptional levels of some differently expressed genes obtained from transcriptome data were further confirmed by qRT-PCR. The results indicated that the inactivation of *vmeL* decreased the relative expression of fourteen T3SS1-related genes, including eight T3SS1 structural genes and six T3SS1 effector gene (Figures 7A,B). On the other hand, the transcriptional levels were complemented in CΔ*vmeL* indicating T3SS1-related genes in *V. parahaemolyticus* are affected by VmeL. But qRT-PCR data showed that *tdh*, which encodes thermostable direct hemolysin, was not altered.

Furthermore, qRT-PCR analysis was used to verify the expression of flagellar-related genes that are shown to be altered in RNA-seq. the results demonstrated that the six polar flagellar assembly–related genes (*VP0790*, *VP2950*, *VP2237*, *VP2256*, *VP2257*, and *VP2259*) were downregulated (Figure 7C) and two lateral flagellar genes (*VPA1540* and *VPA1541*), which encode flagellar motor switch proteins, were downregulated. In addition, RNA samples of strains were collected from swarming plates, and some specific genes were chosen as readouts to reflect the transcriptional level of lateral flagellar genes. The results showed that the transcriptional levels of *flgM*, *flgA*, *flgB*, *motY*, l*afA*, and *fliD* in *V. parahaemolyticus* were significantly downregulated when *vmeL* was deleted (Figure 7D). The transcriptional levels of these lateral flagellar genes, except for *flgM*, were all restored after *vmeL* was complemented.

## Discussion

The purpose of this study was to further investigate the biological contributions of the RND efflux protein VmeL in *V. parahaemolyticus*. We found that *vmeL*-deletion did not affect antimicrobial resistance, which is similar to a previous study that they found that single deletion of *vmeLM* show the same drug susceptibility as the wild type strain *V. parahaemolyticus* AQ334 (Matsuo et al., 2013). Moreover, our results firstly showed that the membrane fusion protein VmeL modulated biofilm formation, motility, and virulence, and possibly affected multiple metabolic pathways in *V. parahaemolyticus*.

Biofilms are aggregates of microorganisms embedded in an extracellular polymeric substances matrix and often adhere to a surface, they are highly tolerant to many antimicrobial agents, including antibiotics, heavy metals, and dyes (Yildiz and Visick, 2009; Flemming et al., 2016). In other bacteria, including *A. nosocomialis* (Knight et al., 2018) and *A. baumannii* (Srinivasan et al., 2015; Kim et al., 2021), researchers found that biofilm formation can be modulated by efflux pumps. In the present study, we discovered similar results that the loss of *vmeL* significantly decreased the biofilm formation of *V. parahaemolyticus* SH112. Similarly, a non-RND membrane fusion protein gene *mfpC* (*VPA1443*) mutant exhibited poor biofilm formation and produced smooth colonies on CR plates (Enos-Berlage et al., 2005). Previous studies have shown that Congo red binding and colony morphology is closely related with the synthesis of exopolysaccharides (Bassis and Visick, 2010; Chen et al., 2010; Wang et al., 2013; Reichhardt et al., 2016). Our results confirmed that the loss of *vmeL* in *V. parahaemolyticus* produced a more translucent colony on the HI plate and exhibited poorer binding ability with Congo red dye compared with WT strains. The exopolysaccharide plays a key role in the maturing of the biofilm (Yildiz and Visick, 2009). In *V. parahaemolyticus*, regulation of exopolysaccharide production and biofilm formation is complex, and involves numerous transcriptional regulators and operons, particularly two component signal transduction, quorum sensing regulators and the *VPA1403-1412* (*cpsA-J*) operon (Boles and McCarter, 2002; Yildiz and Visick, 2009; Zhang et al., 2018). RNA-seq results showed the transcriptional level of *cpsA* is downregulated, while *cpsG* and *cpsJ* were upregulated, in the *vmeL* mutant, compared with wild type. In addition, alterations in gene expressions of quorum sensing and two component systems were found in transcriptomic data. Taken together, we speculated that membrane fusion proteins VmeL could affect biofilm formation in *V. parahaemolyticus* by means of decreasing the amount of extracellular matrix, especially exopolysaccharides. However, it is worth noting that extracellular DNA and proteins are also essential for biofilm formation of *V. parahaemolyticus* (Li et al., 2020). Therefore, we need to further clarify the causation of VmeL could influence biofilm formation of *V. parahaemolyticus.*

Flagellar motility in *vibrio* spp. is associated with several cellular processes, such as movement, colonization, adhesion, biofilm formation, and virulence (Taw et al., 2015; Khan et al., 2020). Surprisingly, swarming motility was eliminated in *V. parahaemolyticus* when *vmeL* was deleted. Swarming is considered a form of flagellum-driven bacterial group motility on a semi-solid surface, that facilitates the acquisition of nutrients and resistance to adverse environmental conditions (Jose and Singh, 2020). Similar results have only been found for the plant pathogen *Pseudomonas syringae* pv. tabaci 6605, in which inner membrane protein *mexF* deletion, abrogated swarming motility (Ichinose et al., 2020). Based on the TEM results, we inferred that VmeL influences the swarming motility of *V. parahaemolyticus* by affecting lateral flagellar assembly. However, in the DEG profile, only two lateral flagellar genes, *VPA1540* and *VPA1541*, were downregulated. A reasonable explanation is that the sample used for RNA sequencing was collected through strains cultivated in MLB medium, but the lateral flagella of *V. parahaemolyticus* are not activated in a fluid environment (McCarter, 2004). Thus, there was no effect on the DEG profile, and the further qRT-PCR analysis affirmed this suspicion. The deletion of *vmeL* when incubated on swarming plates led to reduction in the transcriptional level of *flgM*, *flgA*, *flgB*, *motY*, *lafA*, *fliD*, that could be seen as the readouts of every operon of lateral flagellar genes (Stewart and McCarter, 2003). These results suggested that the swarming repression in the Δ*vmeL* mutant occurred through alterations to lateral flagellar biosynthesis.

Moreover, Swimming motility was abolished when *vmeL* was deleted in *V. parahaemolyticus.*, and a similar phenomenon was observed in *A. nosocomialis* (Knight et al., 2018) and *A. baumannii* (Srinivasan et al., 2015). Although the qRT-PCR results confirmed that some polar flagellar genes were downregulated when *vmeL* was deleted, the TEM results showed that Δ*vmeL* cells exhibited polar flagella. These results suggest that RND efflux pumps can affect the swimming motility of bacteria through affecting the function of flagella rather than depressing flagellar assembly. The basal body, the hook and the filament worked together to structure effective bacterial flagellum, which assembled in a hierarchical manner starting at the inner cytoplasmic membrane and ultimately outside the cell (Echazarreta and Klose, 2019). Interestingly, all components of RND efflux systems are also located in the membrane structure, including membrane fusion proteins that exist in the periplasmic space (Venter et al., 2015). Based on these, we infer that RND efflux systems could interactively function with bacterial polar flagella, thereby led the abolishment of swimming motility.

The pathogenicity of *V. parahaemolyticus* is closely related to a variety of virulence factors, which enable it to cause not only gastroenteritis but also wound infections and septicemia (Daniels et al., 2000; Broberg et al., 2011). Previous studies have proved that the murine intraperitoneal infection model was successfully established and employed to investigate the pathogenicity of *V. parahaemolyticus* (Hiyoshi et al., 2010; Matsuda et al., 2019; Liu et al., 2021). In our study, the virulence, towards HeLa cells and ICR mouse, of *V. parahaemolyticus* was found to be lessened when in a *vmeL* mutant compared with wild type. Bacterial virulence is a sophisticated phenomenon that requires the coordinated expression of various genes types (Lee et al., 2006). Thermostable direct hemolysin (TDH), TDH-related hemolysin (TRH), and type 3 secretion systems (T3SSs) were seen as major pathogenic factors in *V. parahaemolyticus* (Li et al., 2019). T3SS1 mainly accounts for cytotoxicity, while T3SS2 mainly accounts for enterotoxicity (Hiyoshi et al., 2010). In the present work, the results of qRT-PCR displayed that most T3SS1-related genes were downregulated in Δ*vmeL* compared with wild type strains. A similar study found that RND null mutants increased *leuO* transcription in *V. cholera* (Bina et al., 2018), and the *leuO*-homologous protein CalR repressed the transcription of T3SS1 in *V. parahaemolyticus* (Gode-Potratz et al., 2010; Zhang et al., 2021b). Therefore, we inferred that VmeL mediates cytotoxicity by indirectly modulating the transcription of T3SS1 by an unknown mechanism. TDH has been shown to be associated with lethality in the murine intraperitoneal infection model (Matsuda et al., 2019). But inactivation of *vmeL* has no effect on the transcription of *tdh*. In addition, several studies demonstrated that the biofilm formation and surface motility of *V. parahaemolyticus* show a positive correlation with virulence toward mice (Wang et al., 2013; Whitaker et al., 2014; Karan et al., 2021; Li et al., 2022). Consequently, we speculated that VmeL-mediated lethality in the murine intraperitoneal infection on the ground of affecting surface motility, biofilm formation and the transcription of T3SS1-related genes.

RNA-seq results showed that various pathways were altered by *vmeL* deletion, including those related to ABC transporter systems, bacterial secretion systems, two-component systems, quorum sensing, and amino acid metabolism, suggesting that the global transcription in a *vmeL* mutant is disrupted compared with wild type. However, it is important to note that VmeL is an intrinsic membrane fusion protein that needs to be coupled with inner membrane protein VmeM to form an effective RND efflux system (Alvarez-Ortega et al., 2013; Matsuo et al., 2013), rather than a transcriptional regulator. Moreover, membrane fusion protein and inner membrane protein were often found in an operon (Martinez et al., 2009; Matsuo et al., 2013). In addition, qRT-PCR showed that the transcriptional level of *vmeM* dropped about 55% in the *vmeL* mutant compared with wild type, while *vmeM* expression was restored in the CΔ*vmeL* strain (Supplementary Figure 2). These results indicated that *vmeL* positively regulates the transcription of the *vmeLM* operon. In *V. cholerae*, the accumulation of vibriobactin leaded to activation of the stress response Cpx two-component system in the absence of *vexGH* belonging to RND efflux systems (Kunkle et al., 2017). Two-component systems are cell-signaling circuitries that could maintain cellular hemostasis through sensing environmental signals (Papon and Stock, 2019). Similar to our RNA-seq results Bina et al. (2018) found that RND efflux systems had wide-ranging effects on *V. cholerae* transcriptome, such as several two-component systems. Therefore, we inferred that VmeL may affect multiple aspects of bacterial physiology presumable owing to the accumulation of substrates that could activate the two-component systems, which are normally from the periplasm by the VmeLM RND efflux systems. Notably Matsuo et al. (2013) tested the MICs of all 12 efflux systems deletion strains were decreased significantly, but also found most single deletions of RND efflux systems in *V. parahaemolyticus* AQ443 had no effect on drug resistance. This suggested the deficiency of one efflux systems gene deletion could be supplemented by other efflux systems. In our study, we found that *vmeL*-deletion did not affect antimicrobial resistance, and the RNA-seq analysis showed that the transcripts of the other 11 RNA-efflux systems or the outer membrane protein gene *vpoC*, were not impacted by the loss of *vmeL* **(Supplementary Table 2)**. Besides, Δ*vmeL* strains exhibited transcriptional changes in 36 ABC transporter genes, which are similar with RND efflux systems, could efflux various substrates, including antibiotics (Alvarez-Ortega et al., 2013). These results suggested that there is an unknown functional interplay among different RND efflux systems or maybe among different kinds of transporter systems that functions to maintain cellular metabolic homeostasis.

In the present study, we found that VmeL in *V. parahaemolyticus* SH112 influences various biological functions including biofilm formation, swarming motility, swimming motility, and virulence towards HeLa cells and mice. Understanding the roles of VmeL can be useful for developing effective methods to target RND efflux systems to control both contamination and clinical infection caused by *V. parahaemolyticus*. Nevertheless, the mechanism by which VmeL affect various phenotypes have not been fully elucidated, as this will requires extensive experimentation. In view of our findings, we suggest that VmeL of *V. parahaemolyticus* is a potential target for the developments of vaccines to control contamination and infection by *V. parahaemolyticus*.

## Data availability statement

The datasets presented in this study can be found in online repositories. The names of the repository/repositories and accession number(s) can be found below: https://www.ncbi.nlm.nih.gov/, PRJNA797934.

## Ethics statement

All animal infection experiments were approved by the Animal Ethics Committee of the Shanghai Veterinary Research Institute, Chinese Academy of Agricultural Sciences (no. SYXK2020-0027).

## Author contributions

WJ conceived and designed the study. P-XL, X-YZ, YQ, and Y-YL carried out the experiments. P-XL and WJ analyzed the data. P-XL wrote the first draft of the manuscript and all authors contributed to subsequent revisions. All authors read and approved the final manuscript.

## Footnotes

1
https://blast.ncbi.nlm.nih.gov/Blast.cgi

## References

[B1] Alav I. Kobylka J. Kuth M. S. Pos K. M. Picard M. Blair J. M. A. (2021). Structure, assembly, and function of tripartite efflux and type 1 secretion systems in gram-negative bacteria. *Chem. Rev.* 121 5479–5596. 33909410 10.1021/acs.chemrev.1c00055 PMC8277102

[B2] Alvarez-Ortega C. Olivares J. Martinez J. L. (2013). RND multidrug efflux pumps: What are they good for? *Front. Microbiol.* 4:7. 10.3389/fmicb.2013.00007 PMC3564043 23386844

[B3] Banu S. F. Rubini D. Murugan R. Vadivei V. Gowrishankar S. Pandian S. K. (2018). Exploring the antivirulent and sea food preservation efficacy of essential oil combined with DNase on Vibrio parahaemolyticus. *Lwt Food Sci. Technol.* 95 107–115.

[B4] Bassis C. M. Visick K. L. (2010). The cyclic-di-GMP phosphodiesterase BinA negatively regulates cellulose-containing biofilms in *Vibrio fischeri*. *J. Bacteriol.* 192 1269–1278. 10.1128/JB.01048-09 20061475 PMC2820850

[B5] Bina X. R. Howard M. F. Taylor-Mulneix D. L. Ante V. M. Kunkle D. E. Bina J. E. (2018). The *Vibrio cholerae* RND efflux systems impact virulence factor production and adaptive responses via periplasmic sensor proteins. *PLoS Pathog* 14:e1006804. 10.1371/journal.ppat.1006804 29304169 PMC5773229

[B6] Boles B. R. McCarter L. L. (2002). Vibrio parahaemolyticus scrABC, a novel operon affecting swarming and capsular polysaccharide regulation. *J. Bacteriol.* 184 5946–5954. 10.1128/JB.184.21.5946-5954.2002 12374828 PMC135390

[B7] Broberg C. A. Calder T. J. Orth K. (2011). Vibrio parahaemolyticus cell biology and pathogenicity determinants. *Microbes Infect.* 13 992–1001. 21782964 10.1016/j.micinf.2011.06.013 PMC3384537

[B8] Chen Y. Dai J. Morris J. G. Jr. Johnson J. A. (2010). Genetic analysis of the capsule polysaccharide (K antigen) and exopolysaccharide genes in pandemic *Vibrio parahaemolyticus* O3:K6. *BMC Microbiol.* 10:274. 10.1186/1471-2180-10-274 21044320 PMC2987987

[B9] Daniels N. A. Mackinnon L. Bishop R. Altekruse S. Ray B. Hammond R. M. (2000). *Vibrio parahaemolyticus* infections in the united states, 1973-1998. *J. Infect. Dis.* 181 1661–1666. 10823766 10.1086/315459

[B10] Echazarreta M. A. Klose K. E. (2019). Vibrio flagellar synthesis. *Front. Cell Infect. Microbiol.* 9:131. 10.3389/fcimb.2019.00131 PMC6504787 31119103

[B11] Enos-Berlage J. L. Guvener Z. T. Keenan C. E. Mccarter L. L. (2005). Genetic determinants of biofilm development of opaque and translucent *Vibrio parahaemolyticus*. *Mol. Microbiol.* 55 1160–1182. 10.1111/j.1365-2958.2004.04453.x 15686562

[B12] Figurski D. H. Helinski D. R. (1979). Replication of an origin-containing derivative of plasmid RK2 dependent on a plasmid function provided in trans. *Proc. Natl. Acad. Sci. U.S.A.* 76 1648–1652. 10.1073/pnas.76.4.1648 377280 PMC383447

[B13] Flemming H. C. Wingender J. Szewzyk U. Steinberg P. Rice S. A. Kjelleberg S. (2016). Biofilms: An emergent form of bacterial life. *Nat. Rev. Microbiol.* 14 563–575. 27510863 10.1038/nrmicro.2016.94

[B14] Ghenem L. Elhadi N. Alzahrani F. Nishibuchi M. (2017). Vibrio parahaemolyticus: A review on distribution, pathogenesis, virulence determinants and epidemiology. *Saudi J. Med. Med. Sci.* 5 93–103. 30787765 10.4103/sjmms.sjmms_30_17 PMC6298368

[B15] Gode-Potratz C. J. Chodur D. M. Mccarter L. L. (2010). Calcium and iron regulate swarming and type III secretion in vibrio parahaemolyticus. *J. Bacteriol.* 192 6025–6038. 10.1128/JB.00654-10 20851895 PMC2976450

[B16] Gu D. Meng H. M. Li Y. Ge H. J. Jiao X. A. (2019). A GntR family transcription factor (vpa1701) for swarming motility and colonization of *Vibrio parahaemolyticus*. *Pathogens* 8:235. 10.3390/pathogens8040235 31766229 PMC6963403

[B17] Hall C. W. Mah T. F. (2017). Molecular mechanisms of biofilm-based antibiotic resistance and tolerance in pathogenic bacteria. *FEMS Microbiol. Rev.* 41 276–301. 28369412 10.1093/femsre/fux010

[B18] Harshey R. M. Partridge J. D. (2015). Shelter in a swarm. *J. Mol. Biol.* 427 3683–3694. 26277623 10.1016/j.jmb.2015.07.025 PMC4548829

[B19] Hiyoshi H. Kodama T. Iida T. Honda T. (2010). Contribution of *Vibrio parahaemolyticus* virulence factors to cytotoxicity, enterotoxicity, and lethality in mice. *Infect. Immun.* 78 1772–1780. 10.1128/IAI.01051-09 20086084 PMC2849405

[B20] Hotinger J. A. Morris S. T. May A. E. (2021). The case against antibiotics and for anti-virulence therapeutics. *Microorganisms* 9:2049. 10.3390/microorganisms9102049 PMC8537500 34683370

[B21] Ichinose Y. Nishimura T. Harada M. Kashiwagi R. Yamamoto M. Noutoshi Y. (2020). Role of two sets of RND-type multidrug efflux pump transporter genes, mexAB-oprM and mexEF-oprN, in virulence of *Pseudomonas* syringae pv. tabaci 6605. *Plant Pathol. J.* 36 148–156. 10.5423/PPJ.OA.11.2019.0273 32296294 PMC7143514

[B22] Jose R. Singh V. (2020). Swarming in bacteria: A tale of plasticity in motility behavior. *J. Indian Instit. Sci.* 100 515–524.

[B23] Karan S. Garg L. C. Choudhury D. Dixit A. (2021). Recombinant FimH, a fimbrial tip adhesin of *Vibrio parahaemolyticus*, elicits mixed T helper cell response and confers protection against *Vibrio parahaemolyticus* challenge in murine model. *Mol. Immunol.* 135 373–387. 10.1016/j.molimm.2021.05.005 34020083

[B24] Khan F. Tabassum N. Anand R. Kim Y. M. (2020). Motility of vibrio spp.: Regulation and controlling strategies. *Appl. Microbiol. Biotechnol.* 104 8187–8208. 10.1007/s00253-020-10794-7 32816086

[B25] Kim C. M. Park G. Ko Y. J. Kang S. H. Jang S. J. (2021). Relationships between relative expression of RND efflux pump genes, H33342 efflux activity, biofilm-forming activity, and antimicrobial resistance in *Acinetobacter baumannii* clinical isolates. *JPN J. Infect. Dis.* 74, 499–506. 10.7883/yoken.JJID.2020.765 33642430

[B26] Knight D. B. Rudin S. D. Bonomo R. A. Rather P. N. (2018). Acinetobacter nosocomialis: defining the role of efflux pumps in resistance to antimicrobial therapy, surface motility, and biofilm formation. *Front. Microbiol.* 9:1902. 10.3389/fmicb.2018.01902 30186249 PMC6111201

[B27] Kunkle D. E. Bina X. R. Bina J. E. (2017). The *Vibrio cholerae* VexGH RND efflux system maintains cellular homeostasis by effluxing vibriobactin. *mBio* 8 e00126–17. 10.1128/mBio.00126-17 28512090 PMC5433094

[B28] Lee D. G. Urbach J. M. Wu G. Liberati N. T. Feinbaum R. L. Miyata S. (2006). Genomic analysis reveals that *Pseudomonas aeruginosa* virulence is combinatorial. *Geno. Biol.* 7:R90. 10.1186/gb-2006-7-10-r90 17038190 PMC1794565

[B29] Lei T. Jiang F. He M. Zhang J. Zeng H. Chen M. (2020). Prevalence, virulence, antimicrobial resistance, and molecular characterization of fluoroquinolone resistance of *Vibrio parahaemolyticus* from different types of food samples in China. *Int. J. Food. Microbiol.* 317:108461. 10.1016/j.ijfoodmicro.2019.108461 31794931

[B30] Li L. Meng H. Gu D. Li Y. Jia M. (2019). Molecular mechanisms of *Vibrio parahaemolyticus* pathogenesis. *Microbiol. Res.* 222 43–51. 30928029 10.1016/j.micres.2019.03.003

[B31] Li W. Wang J. J. Qian H. Tan L. Zhang Z. Liu H. (2020). Insights into the role of extracellular DNA and extracellular proteins in biofilm formation of *Vibrio parahaemolyticus*. *Front. Microbiol.* 11:813. 10.3389/fmicb.2020.00813 32508761 PMC7248202

[B32] Li Y. Sun W. Wang Q. Yu Y. Wan Y. Zhou K. (2022). The GntR-like transcriptional regulator HutC involved in motility, biofilm-forming ability, and virulence in *Vibrio parahaemolyticus*. *Microb Pathog* 167 105546. 10.1016/j.micpath.2022.105546 35512440

[B33] Lian L. Xue J. Li W. Ren J. Tang F. Liu Y. (2021). VscF in T3SS1 helps to translocate VPA0226 in *Vibrio parahaemolyticus*. *Front. Cell Infect. Microbiol.* 11:652432. 10.3389/fcimb.2021.652432 33869083 PMC8047418

[B34] Liu J. F. Qin K. W. Wu C. L. Fu K. F. Yu X. J. Zhou L. J. (2021). De novo sequencing provides insights into the pathogenicity of foodborne *Vibrio parahaemolyticus*. *Front. Cell. Infect. Microbiol.* 11:652957. 10.3389/fcimb.2021.652957 34055666 PMC8162212

[B35] Livak K. J. Schmittgen T. D. (2001). Analysis of relative gene expression data using real-time quantitative PCR and the 2(-delta delta C(T)) method. *Methods* 25 402–408. 11846609 10.1006/meth.2001.1262

[B36] Martinez J. L. Sanchez M. B. Martinez-Solano L. Hernandez A. Garmendia L. Fajardo A. (2009). Functional role of bacterial multidrug efflux pumps in microbial natural ecosystems. *FEMS Microbiol. Rev.* 33 430–449. 19207745 10.1111/j.1574-6976.2008.00157.x

[B37] Matsuda S. Okada R. Tandhavanant S. Hiyoshi H. Gotoh K. Iida T. (2019). Export of a *Vibrio parahaemolyticus* toxin by the sec and type III secretion machineries in tandem. *Nat. Microbiol.* 4 781–788. 10.1038/s41564-019-0368-y 30778145

[B38] Matsuo T. Hayashi K. Morita Y. Koterasawa M. Ogawa W. Mizushima T. (2007). VmeAB, an RND-type multidrug efflux transporter in *Vibrio parahaemolyticus*. *Microbiology (Reading)* 153 4129–4137. 10.1099/mic.0.2007/009597-0 18048926

[B39] Matsuo T. Nakamura K. Kodama T. Mikami T. Hiyoshi H. Tsuchiya T. (2013). Characterization of all RND-type multidrug efflux transporters in *Vibrio parahaemolyticus*. *Microbiologyopen* 2 725–742. 10.1002/mbo3.100 23894076 PMC3831635

[B40] McCarter L. (1999). The multiple identities of *Vibrio parahaemolyticus*. *J. Mol. Microbiol. Biotechnol.* 1 51–57. 10941784

[B41] McCarter L. L. (2004). Dual flagellar systems enable motility under different circumstances. *J. Mol. Microbiol. Biotechnol.* 7 18–29. 10.1159/000077866 15170400

[B42] Morales V. M. Backman A. Bagdasarian M. (1991). A series of wide-host-range low-copy-number vectors that allow direct screening for recombinants. *Gene* 97 39–47. 10.1016/0378-1119(91)90007-x 1847347

[B43] Ono T. Park K. S. Ueta M. Iida T. Honda T. (2006). Identification of proteins secreted via *Vibrio parahaemolyticus* type III secretion system 1. *Infect. Immun.* 74 1032–1042. 10.1128/IAI.74.2.1032-1042.2006 16428750 PMC1360304

[B44] Ottaviani D. Leoni F. Serra R. Serracca L. Decastelli L. Rocchegiani E. (2012). Nontoxigenic *Vibrio parahaemolyticus* strains causing acute gastroenteritis. *J. Clin. Microbiol.* 50 4141–4143. 10.1128/JCM.01993-12 23052317 PMC3502970

[B45] Papon N. Stock A. M. (2019). Two-component systems. *Curr. Biol.* 29 R724–R725. 31386843 10.1016/j.cub.2019.06.010

[B46] Reichhardt C. Mccrate O. A. Zhou X. Lee J. Thongsomboon W. Cegelski L. (2016). Influence of the amyloid dye congo red on curli, cellulose, and the extracellular matrix in E. coli during growth and matrix purification. *Anal. Bioanal. Chem.* 408 7709–7717. 10.1007/s00216-016-9868-2 27580606

[B47] Srinivasan V. B. Venkataramaiah M. Mondal A. Rajamohan G. (2015). Functional characterization of AbeD, an RND-type membrane transporter in antimicrobial resistance in *Acinetobacter baumannii*. *PLoS One* 10:e0141314. 10.1371/journal.pone.0141314 26496475 PMC4619830

[B48] Stewart B. J. McCarter L. L. (2003). Lateral flagellar gene system of *Vibrio parahaemolyticus*. *J. Bacteriol.* 185 4508–4518. 10.1128/JB.185.15.4508-4518.2003 12867460 PMC165745

[B49] Su Y. C. Liu C. (2007). *Vibrio parahaemolyticus*: A concern of seafood safety. *Food Microbiol.* 24 549–558. 10.1016/j.fm.2007.01.005 17418305

[B50] Tandhavanant S. Matsuda S. Hiyoshi H. Iida T. Kodama T. (2018). Vibrio parahaemolyticus senses intracellular K+ to translocate type III secretion system 2 effectors effectively. *mBio* 9:e01366–18. 10.1128/mBio.01366-18 30042203 PMC6058294

[B51] Taw M. Lee H. I. Lee S. H. Chang W. S. (2015). Characterization of MocR, a GntR-like transcriptional regulator, in *Bradyrhizobium japonicum*: Its impact on motility, biofilm formation, and soybean nodulation. *J. Microbiol.* 53 518–525. 10.1007/s12275-015-5313-z 26224454

[B52] Uddin T. M. Chakraborty A. J. Khusro A. Zidan B. R. M. Mitra S. Emran T. B. (2021). Antibiotic resistance in microbes: History, mechanisms, therapeutic strategies and future prospects. *J. Infect. Public Health.* 14, 1750–1766. 10.1016/j.jiph.2021.10.020 34756812

[B53] Venter H. Mowla R. Ohene-Agyei T. Ma S. (2015). RND-type drug e ffl ux pumps from gram-negative bacteria: molecular mechanism and inhibition. *Front. Microbiol.* 6:377. 10.3389/fmicb.2015.00377 25972857 PMC4412071

[B54] Wadhwa N. Berg H. C. (2021). Bacterial motility: Machinery and mechanisms. *Nat. Rev. Microbiol.* 20, 161–173. 34548639 10.1038/s41579-021-00626-4

[B55] Wang L. Ling Y. Jiang H. Qiu Y. Qiu J. Chen H. (2013). AphA is required for biofilm formation, motility, and virulence in pandemic *Vibrio parahaemolyticus*. *Int. J. Food Microbiol.* 160 245–251. 10.1016/j.ijfoodmicro.2012.11.004 23290231

[B56] Whitaker W. B. Richards G. P. Boyd E. F. (2014). Loss of sigma factor RpoN increases intestinal colonization of vibrio parahaemolyticus in an adult mouse model. *Infect. Immun.* 82 544–556. 10.1128/IAI.01210-13 24478070 PMC3911383

[B57] Yildiz F. H. Visick K. L. (2009). Vibrio biofilms: So much the same yet so different. *Trends Microbiol.* 17 109–118. 10.1016/j.tim.2008.12.004 19231189 PMC2729562

[B58] Yu M. J. Zheng L. Wang X. B. Wu M. F. Qi M. Fu W. D. (2019). Comparative transcriptomic analysis of surf clams (*Paphia undulate*) infected with two strains of *Vibrio* spp. reveals the identity of key immune genes involved in host defense. *BMC Geno.* 20:988. 10.1186/s12864-019-6351-4 31847806 PMC6915886

[B59] Yu Y. Yang H. Li J. Zhang P. Wu B. Zhu B. (2012). Putative type VI secretion systems of *Vibrio parahaemolyticus* contribute to adhesion to cultured cell monolayers. *Arch. Microbiol.* 194 827–835. 10.1007/s00203-012-0816-z 22535222

[B60] Zhang L. Weng Y. Wu Y. Wang X. Yin Z. Yang H. (2018). H-NS is an activator of exopolysaccharide biosynthesis genes transcription in *Vibrio parahaemolyticus*. *Microb Pathog* 116 164–167. 10.1016/j.micpath.2018.01.025 29366862

[B61] Zhang Y. Qiu Y. Gao H. Sun J. Li X. Zhang M. (2021a). OpaR controls the metabolism of c-di-GMP in *Vibrio parahaemolyticus*. *Front. Microbiol.* 12:676436. 10.3389/fmicb.2021.676436 34163453 PMC8215210

[B62] Zhang Y. Qiu Y. Xue X. Zhang M. Sun J. Li X. (2021b). Transcriptional regulation of the virulence genes and the biofilm formation associated operons in *Vibrio parahaemolyticus*. *Gut. Pathog.* 13:15. 10.1186/s13099-021-00410-y 33653369 PMC7923509

