# Supplementary material for: Biological and transcriptional studies reveal VmeL is involved in motility, biofilm formation and virulence in Vibrio parahaemolyticus
Source: Front Microbiol. 2022 Aug 9;13:976334. doi: 10.3389/fmicb.2022.976334 (PMC9397117; doi:10.3389/fmicb.2022.976334)
Supplement: Supplementary file 1 [file Data_Sheet_1.docx]

Supplementary material

Frontier in Microbiology

**Biological and transcriptional studies reveal VmeL is involved in motility, biofilm formation and virulence in *Vibrio parahaemolyticus***

**Peng-xuan Liu^1,2^, Xiao-yun Zhang^1^, Quan Wang^1^, Yang-yang Li^1,2^, Wei-dong Sun^2^, Yu Qi^1^, Kai Zhou^3^, Xian-gan Han^1^,** **Zhao-guo Chen^1^, Wei-huan Fang^4^, Wei Jiang^1*^**

1 Shanghai Veterinary Research Institute, Chinese Academy of Agricultural Sciences, Shanghai 200241, PR China

2 Nanjing Agricultural University, Nanjing, PR China

3 Shenzhen Institute of Respiratory Diseases, the First Affiliated Hospital (Shenzhen People's Hospital), Shenzhen, China

4 Institute of Preventive Veterinary Medicine and Zhejiang Provincial Key Laboratory of Preventive Veterinary Medicine, Zhejiang University, Hangzhou, PR China

**∗ Corresponding author:**

Wei Jiang, Shanghai Veterinary Research Institute, Chinese Academy of Agricultural Sciences, No. 518, Ziyue Road, Minhang District, Shanghai 200241, China. Tel/fax: +86 21 34293397.

Email: jiangweijw99@163.com

**A**


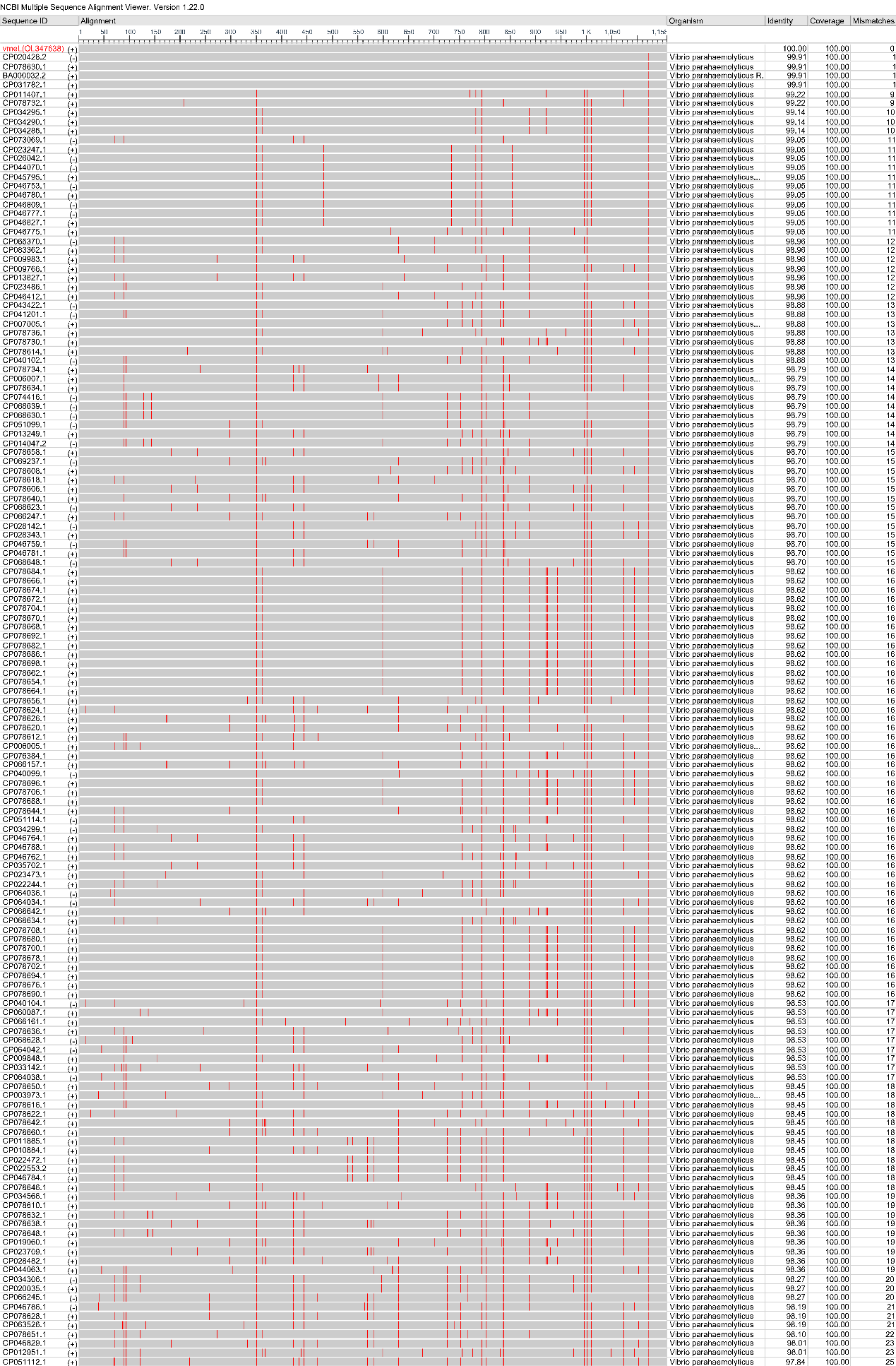


**B**


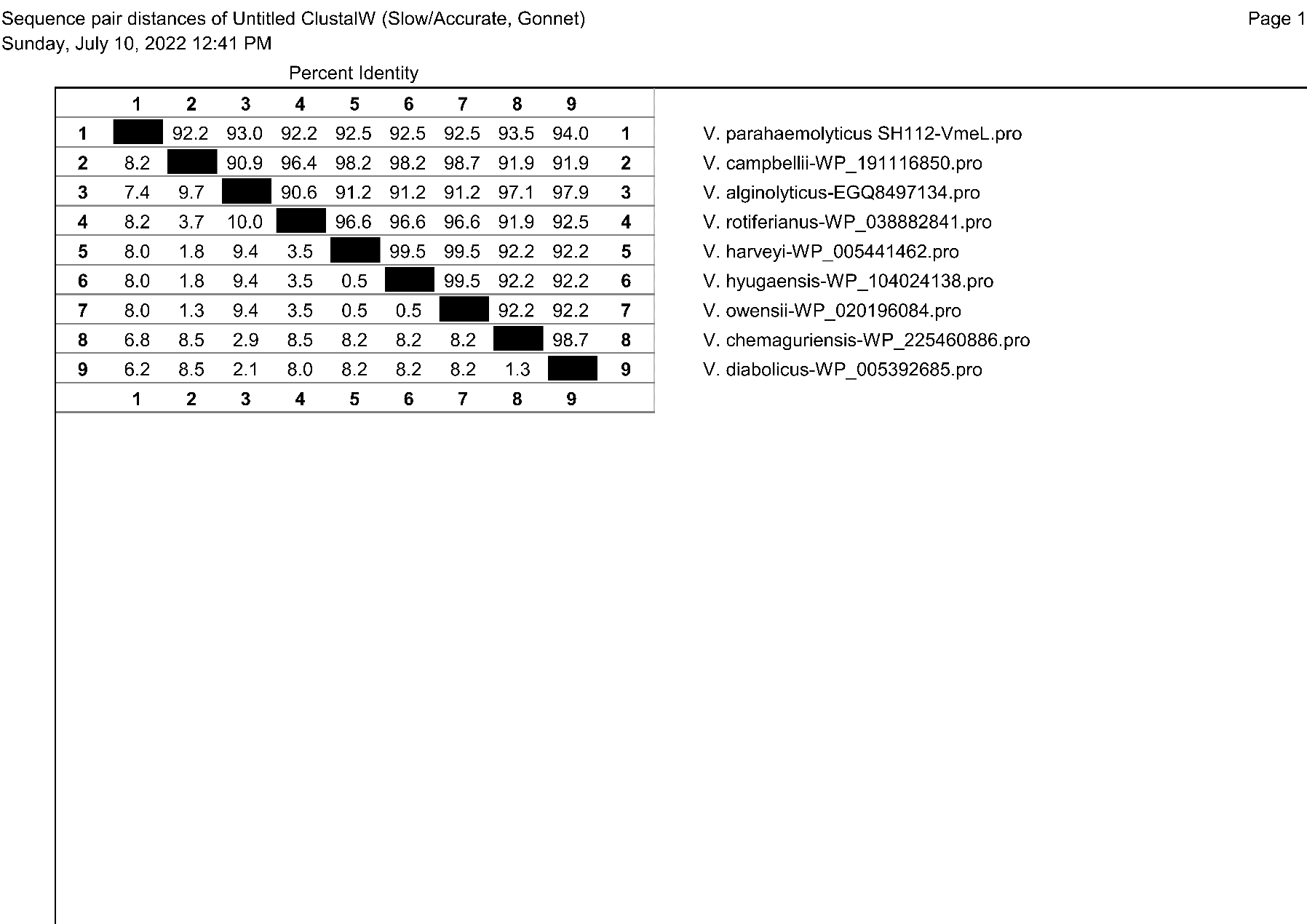


**Supplementary Figure 1**. **(A)** the multiple alignment of *vmeL* (GenBank: OL347638) of *V. parahaemolyticus* SH112 was constructed using NCBI (National Center for Biotechnology Information) Multiple Sequence Alignment Viewer (version 1.22.0). a total of 143 *V. parahaemolyticus* isolates were identified for this analysis **(B)** Homologous analysis of VmeL in *V. parahaemolyticus* SH112. The amino acid sequences of VmeL were translated from nucleotide sequence of *vmeL*(GenBank: OL347638), and the amino acid sequences of other homologous proteins were downloaded from NCBI database. The sequence pair distances of ClustalW (Slow/Accurate, Gonnet) was constructed using MegAlign software.

**B**

**A**


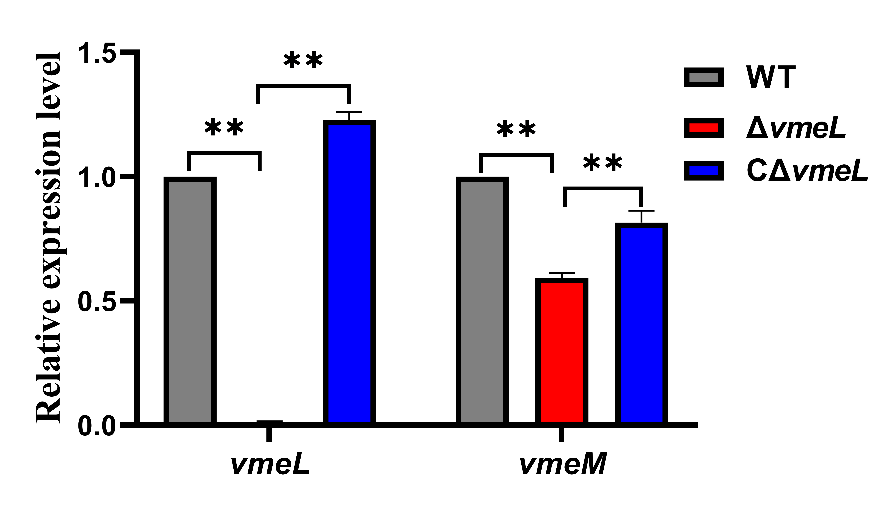

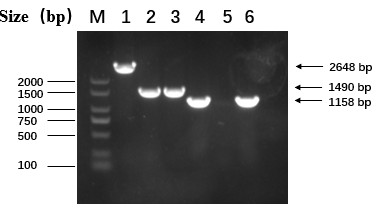


**Supplementary Figure 2**. **(A)** PCR identification of WT, Δ*vmeL*, CΔ*vmeL*. M: DL 2000; lane 1–3: Primers of *vmeL*-E/F; lane 4–6: Primers of pMMB-*vmeL*-F/R; template of lane 1-3 is WT, Δ*vmeL*, CΔ*vmeL* separately; template of lane 4-6 is WT, Δ*vmeL*, CΔ*vmeL*. Separately. **(B)** Quantitative RT-PCR for the relative expression level of *vmeL* and *vmeM* in different strains. ** indicates statistical significance of *P* < 0.01 compared with the WT.


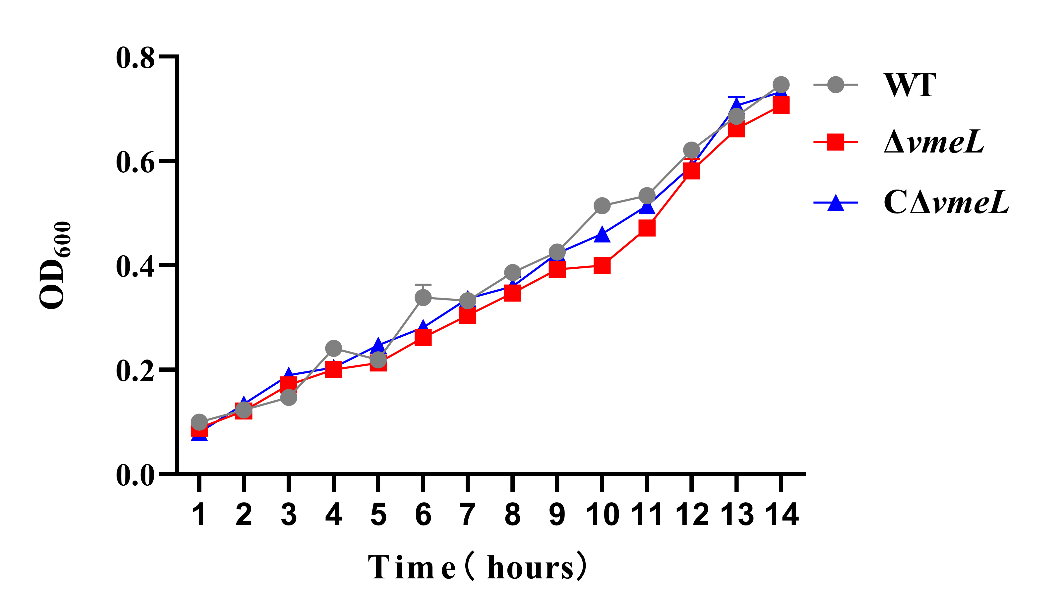


**A**

**B**


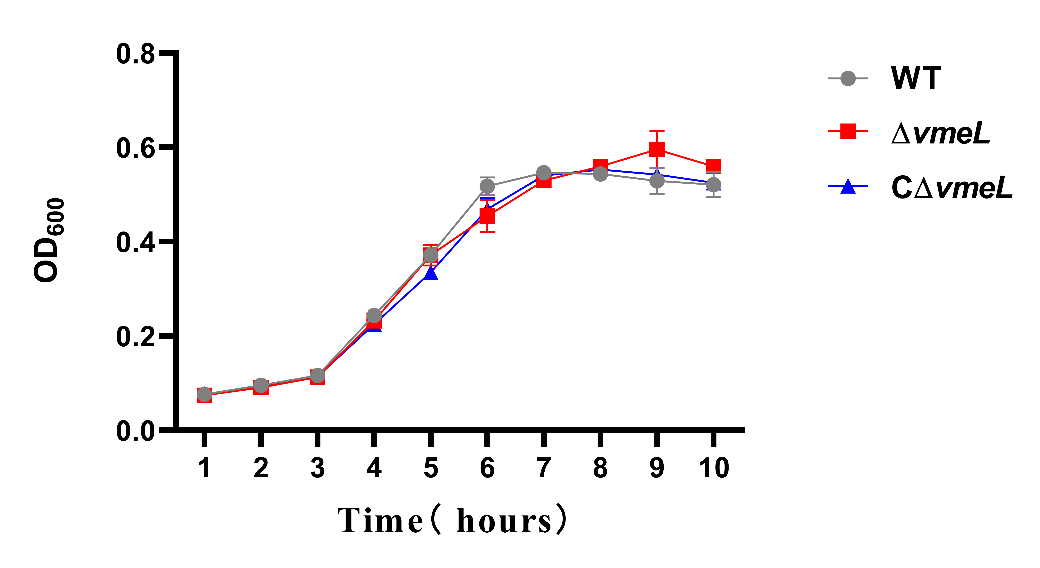


**Supplementary Figure 3**. Growth of *Vibrio parahaemolyticus* WT, Δ*vmeL*, and CΔ*vmeL* in MLB (Luria-Bertani medium with 2% sodium chloride) **(A)**, and in DMEM + 10% FBS **(B)**, at 37 °C with constant shaking at 180 rpm

**
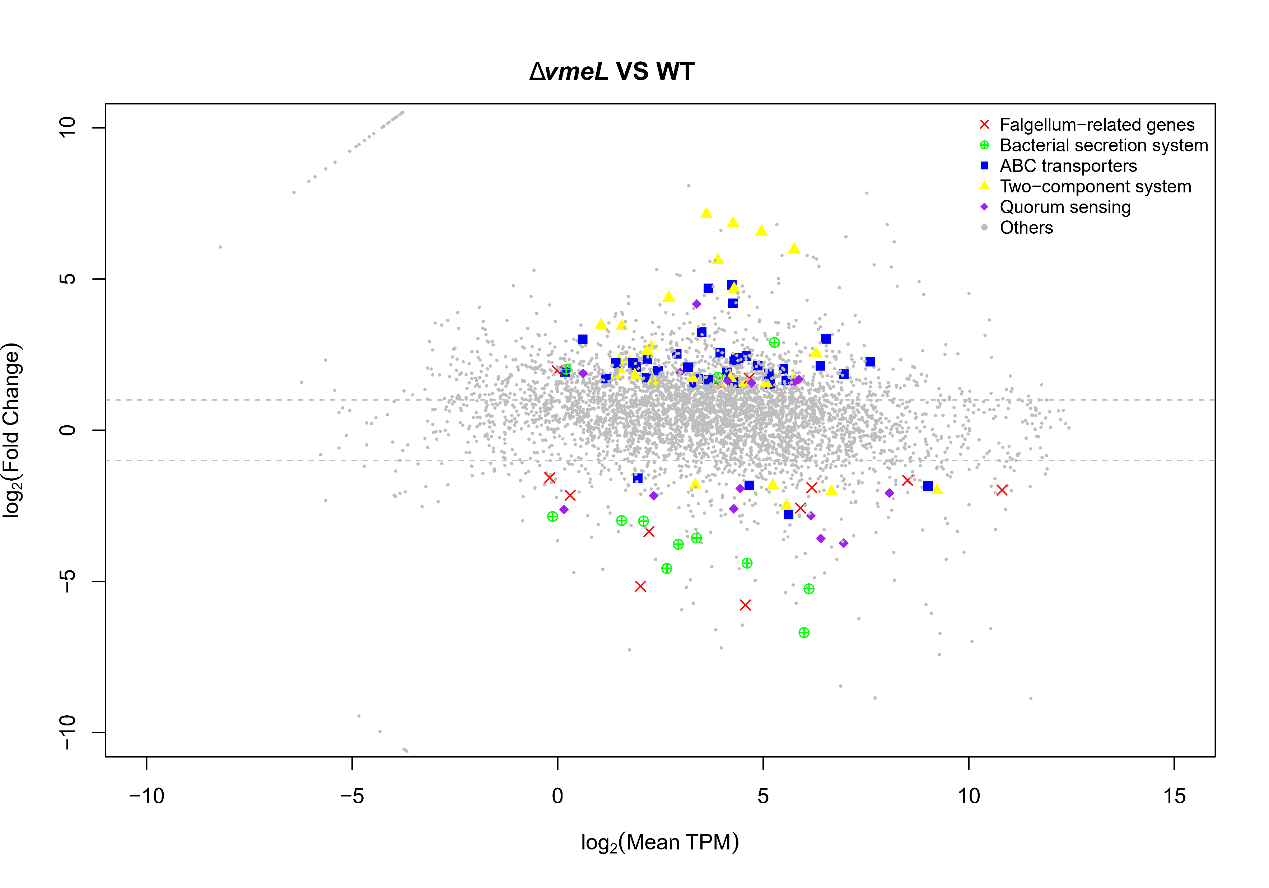
**

**Supplementary Figure 4**. MA plot shows the genes regulated by VmeL. The X-axis (A) represents the logarithm-transformed value of gene expression levels. The Y-axis (M) represents the logarithm-transformed value of expression change folds. TPM indicates transcripts per kilobase of exon model per million mapped reads.

**Supplementary Table 1.** RT-PCR Primers used in this study.

| **Primers** | **Sequence (5' to 3')** |
| --- | --- |
| *VP2237*-RT-F | TCATCTGCTCTGCGATGGTC |
| *VP2237*-RT-R | GCCGTACGTTCGTATCTCGT |
|  |  |
| *VP2256*-RT-F | GTCGATGCGTTGCTGCTTAG |
| *VP2256*-RT-R | GATGTCTGGCGGCATGGATA |
|  |  |
| *VP2257*-RT-F | CGATTGCCGCTTGAACAGAG |
| *VP2257*-RT-R | CACAGCAAGTTCAACCGGTG |
|  |  |
| *VP2259*-RT-F | TGAGTTTGGCGCTTGTTTCG |
| *VP2259*-RT-R | GCGAAAGAAACGACGGCAAT |
|  |  |
| *VP1656*-RT-F | GAAGCCAACGTTGCGAGTTT |
| *VP1656*-RT-R | CTTTGAACGAACCAAGCGCA |
|  |  |
| *VP1667*-RT-F | AGTCCACCGCAGTAGAGACT |
| *VP1667*-RT-R | TCGACACCGAAATCCGTGTT |
|  |  |
| *VP1668*-RT-F | CTGCGTGAAATGTTGGCGAA |
| *VP1668*-RT-R | TCATTGACCTGCGATGCCTT |
|  |  |
| *VP1674*-RT-F | ACCACTTGGCCAATACTCGG |
| *VP1674*-RT-R | AGAAAACGTTGAGTTGCGGC |
|  |  |
| *VP1684*-RT-F | TGGAACAGTTGCACCCAAGT |
| *VP1684*-RT-R | TTTTGTATCGCGAAACGGGC |
|  |  |
| *VP1687*-RT-F | TGACCAAGACACGGCAATCA |
| *VP1687*-RT-R | AAAACCGACTTCCTCGTCCC |
|  |  |
| *VP1688*-RT-F | ATGTCGATGTGGTTGCCGAT |
| *VP1688*-RT-R | CGCCTGAATCTGTCCATCGA |
|  |  |
| *VPA0450*-RT-F | TCGATGGCATGAACCTGCTT |
| *VPA0450*-RT-R | CATGTTTCGTCCACCGATGC |
|  |  |
| *VP2950*-RT-F | CACCAAGTTGGGCTGAAACG |
| *VP2950*-RT-R | CGCTAGGTCTTGTTGGCTCA |
|  |  |
| *vopZ*-RT-F | AGGATGAGGATGAGGAGTGTGA |
| *vopZ*-RT-R | GCAGCGTCAGAACAATCTCG |
|  |  |
| *vopQ*-RT-F | CGCCGATAGCAAAAGAAGCC |
| *vopQ*-RT-R | ATTTCCAAACGTGGCAGCAC |
|  |  |
| *vopR*-RT-F | TCACCGCCACAGAAGATGAC |
| *vopR*-RT-R | TGCCACTGCCTACTCTTTGG |
|  |  |
| *VP1686*-RT-F | GGCAAACTCAGCATTGGTGG |
| *VP1686*-RT-R | TTGCAAACGCTGAGCTTGTC |
|  |  |
| *tdh*-RT-F | CACGTGTGGTCACTGTCGTA |
| *tdh*-RT-R | TGGGATCTTGAACGCGTTGA |
|  |  |
| *vmeL*-RT-F | CTTTTACCCTTCGTTGTCGTCG |
| *vmeL*-RT-R | GGTTCGGTTATGGTTTCTTTTGTTG |
|  |  |
| *vmeM*-RT-F | CATTGGCGTTGGACTGTATTTG |
| *vmeM*-RT-R | GTCGGTACTTTCGGGTTAGGG |
|  |  |
| *gapA*-RT-F | TTTGAAGAGCGTCCGTTGGTG |
| *gapA*-RT-R | TTAAGCCAACACAACGTTACG |
|  |  |
| *flgM*-RT-F | TCTGCTTGGGCACGATCAAT |
| *flgM*-RT-R | GCGGTCATGTCCCACAAACA |
|  |  |
| *flgA*-RT-F | ACTGGTTGGACTTGGCTACG |
| *flgA*-RT-R | ACCTTCTCATCGGAGGAGCT |
|  |  |
| *flgB*-RT-F | TGGGTACTTGGCGAAAGACC |
| *flgB*-RT-R | AATACTGGGCCTGAACCTGC |
|  |  |
| *motY*-RT-F | TCCAACTACCACCGGCAATC |
| *motY*-RT-R | TGGCAAGGTGCGACACTAAT |
|  |  |
| *VPA1540*-RT-F | ACAGGAAAGAATCAGCCGCA |
| *VPA1540*-RT-R | CGTCTGATTGCAGCTCAAGC |
|  |  |
| *VPA1541*-RT-F | TGAACGGTTCCCTACTTGGC |
| *VPA1541*-RT-R | TGCCGAATCGTGTACGTCAA |
|  |  |
| *lafA*-RT-F | GGCATGTCTGTAGCAATGCG |
| *lafA*-RT-R | TGAACTCAGCGTCCATTGCT |
|  |  |
| *fliD*-RT-F | ATGCCAGCAGATCTAGACGC |
| *fliD*-RT-R | CACTCACCGTCGTTACACCA |
|  |  |
| *VP0790*-RT-F | AGACATGCGCTCAGACAACA |
| *VP0790*-RT-R | AGGTCGTTTTTGCCTTGTGC |
|  |  |

**Supplementary Table 2.** RNA-seq results of RND efflux genes except *vmeLM.*

| **Gene-id** | **Name** | **log2FoldChange** | **pValue** | **qValue** |
| --- | --- | --- | --- | --- |
| VP_RS05315 | *vmeA* | -1.20513 | 5.42E-06 | 3.39E-05 |
| VP_RS05320 | *vmeB* | -0.44047 | 0.013025 | 0.032503 |
| VP_RS00205 | *vmeC* | 0.825403 | 0.308751 | 0.437031 |
| VP_RS00200 | *vmeD* | 0.51094 | 0.39414 | 0.52661 |
| VP_RS04610 | *vmeE* | 0.868392 | 0.435136 | 0.567844 |
| VP_RS04615 | *vmeF* | 1.497119 | 0.000803 | 0.002918 |
| VP_RS05715 | *vmeG* | -0.0633 | 0.108711 | 0.19191 |
| VP_RS05720 | *vmeH* | 0.291564 | 0.642685 | 0.747875 |
| VP_RS05725 | *vmeI* | 0.512168 | 0.901343 | 0.94047 |
| VP_RS12005 | *vmeJ* | 0.279029 | 0.341241 | 0.47227 |
| VP_RS12000 | *vmeK* | 1.032765 | 0.230321 | 0.347891 |
| VP_RS16980 | *vpoM* | 0.312685 | 0.253232 | 0.374588 |
| VP_RS16990 | *vmeN* | 1.733061 | 0.243766 | 0.363914 |
| VP_RS16985 | *vmeO* | 1 | 0.495668 | 0.622454 |
| VP_RS17485 | *vmeP* | -0.66742 | 0.439031 | 0.570374 |
| VP_RS17490 | *vmeQ* | 0.611729 | 0.692439 | 0.78654 |
| VP_RS17540 | *vmeR* | 0.168754 | 0.19065 | 0.299189 |
| VP_RS17535 | *vmeS* | 0.888639 | 0.862707 | 0.916553 |
| VP_RS19045 | *vmeT* | 1.175318 | 0.387262 | 0.519917 |
| VP_RS19050 | *vmeU* | 1.39945 | 0.12676 | 0.216958 |
| VP_RS19055 | *vmeV* | 1.771299 | 0.028355 | 0.062856 |
| VP_RS19695 | *vmeW* | -0.34494 | 0.009691 | 0.025224 |
| VP_RS19690 | *vmeX* | -0.57065 | 0.002062 | 0.006674 |
| VP_RS20865 | *vmeY* | 0.772784 | 0.69379 | 0.787691 |
| VP_RS20860 | *vmeZ* | 0.37709 | 0.248148 | 0.36894 |
| VP_RS02035 | *vpoC* | 0.665543 | 0.779776 | 0.85493 |
